# Numerical investigation of the effect of spanwise length and mesh density on flow around cylinder at Re = 3900 using LES model

**DOI:** 10.1371/journal.pone.0266065

**Published:** 2022-04-08

**Authors:** Haider Ali, Niaz Bahadur Khan, Muhammad Jameel, Azam Khan, Muhammad Sajid, Adnan Munir, A. El-Sayed Ahmed, Khalid Abdulkhaliq M. Alharbi, Ahmed M. Galal

**Affiliations:** 1 National University of Sciences and Technology (NUST), Islamabad, Pakistan; 2 Department of Civil Engineering, College of Engineering, King Khalid University, Abha, Saudi Arabia; 3 Mathematics Department, Faculty of Science, Taif University, Taif, Saudi Arabia; 4 Mechanical Engineering Department, College of Engineering, Umm Al-Qura University, Makkah, KSA; 5 Mechanical Engineering Department, College of Engineering, Prince Sattam Bin Abdulaziz University, Wadi addawaser, Saudi Arabia; 6 Production Engineering and Mechanical Design Department, Faculty of Engineering, Mansoura University, Mansoura, Egypt; Fuzhou University, CHINA

## Abstract

Flow around circular cylinder has been extensively studied by researchers for several decades due to its wide range of engineering applications such as in heat exchangers, marine cables, high rise building, chimneys, and offshore structures. The lack of clear understanding of the unsteady flow dynamics in the wake of circular cylinder and high computational cost are still an area of high interest amongst the researchers. The aim of the current study is to investigate the effect of variation in spanwise length and grid resolution in the spanwise direction on the recirculation length, separation angle of wake flow by performing large eddy simulations (LES). This study is an extension to previous work by Khan, NB et al, 2019 in which the spanwise length is restricted to 4D only. In current study, the spanwise length is changed from 0.5D to 8D where D is diameter of cylinder and mesh resolution in the spanwise direction is changed from 1 to 80 elements in the present study. The recirculation length, separation angle and wake characteristics are analyzed in detail. It is concluded that after getting optimize spanwise length, mesh resolution in the spanwise direction is the only parameter contributing toward better result.

## Introduction

Investigating the unsteady nature of flow around cylinder is one of the highly research topics in the field of offshore engineering and fluid-structure interaction (FSI). This unsteady nature is very sensitive in terms of flow separation, boundary layer, wake region characteristics, drag coefficient and Strouhal number. Primarily, the analysis has been performed using the three main turbulent model i.e., direct numerical simulation (DNS), Reynolds-averaged Numerical simulation (RANS) model, and large eddy simulation (LES) model. Each of these models differs in terms of solving the Navier-Stokes equation and presenting the effects of the flow. DNS is the only numerical method in which all spatial and temporal scales of turbulence are resolved in case of flow around a cylinder. However, numerical cost is the major obstacle in using DNS method. RANS model unable to capture the transitional flow characteristics, boundary layer and separation region accurately [1, 2]. Due to limitation of DNS and RANS model, LES is most attractive option for investigating the unsteady nature of flow around cylinder in subcritical regime. The flow around a fixed cylinder at Re = 3900 come under the lower subcritical flow regime, which is highly sensitive in terms of flow separation, boundary layer nature, recirculation length, wake characteristics, and other hydrodynamic coefficients [3]. The availability of large-scale experimental and numerical data [4–10] for the flow around cylinders at Re = 3900 makes it an excellent benchmark case for assessing the capability of computational tools and process.

Breuer [11] performed large eddy simulations at Re = 3900 and investigated the effect of near-wall modeling, sub-grid scale modeling and spanwise resolution on the accuracy of numerical model. In another study Breuer [11] computed the low recirculation length (of less than 1.1) with a Smagorinsky model and comparatively high value of recirculation length when using the dynamic model. Unlike dynamic Smagorinsky model, the traditional Smagorinsky model over-predicted the importance of drag coefficient and separation angle. While systematically investigating the accuracy of LES model on flow past a cylinder at Re = 3900, [11] found that the resolution in spanwise direction effects the three-dimensionality of flow and hence, accuracy of results significantly. Tremblay et al. [12] studied the effects of the SGS model and grid resolution on large eddy simulations using technique known as cartesian grid. The length of recirculation and profiles of mean velocity could not be reliably predicted in this analysis. Lysenko et al. [13] used OpenFOAM tool to investigate the flow aroud a cylinder using a dynamic k-equation SGS model and the large eddy simulations model. Parnaudeau et al. [14] used LES with a high order scheme to simulate a flow around cylinder. The numerical analysis resulted in power spectra and turbulence statistics up to 10 D. Moreover, Wissink and Rodi, Dong et al. [15, 16] used direct numerical simulations (DNS) to investigate flow around cylinder.

Dong et al. [16] and MA, Karamanos, and Karniadakis [17] studied flow around cylinder with spanwise length of 10D using Direct Numerical simulations. At a moderate to high values of Reynolds number, Rajani et al. [2] investigated the limitation of URANS in measuring drag forces, skin friction coefficients and mean pressure coefficients. Zhang et al. [18] investigated the both the effects of infinite and finite cylinders on flow characteristics and observed that free end of cylinder has significant impact on wake characteristics. Wissink et al. [15, 19] used direct numerical simulations in order to run a series of simulations at Reynolds number = 3300 and compared the findings to experimental studies at Reynolds number = 3900. The roll-up of the splitting shear layer, which transformed to turbulence, was observed. Even at low Reynolds numbers, direct numerical simulations are costly and provides accurate and reliable performance. Due to the deficiencies in the URANS method and the high computational cost of direct numerical simulations, large eddy simulations (smagorinsky model) with fixed coefficient but without model, and with a dynamic model is most attractive choice to analyze the unsteady nature of flow around cylinder.

Franke and Frank [20] used a cell-centered volume code to perform a sequence of large eddy simulation at Reynolds number = 3900. The study concluded that small value of recirculation length computed during analysis is mainly due to short-time averaging data. Kravchenko and Moin [21] used large eddy simulations and a high-order precise numerical model which is based on B-splines to investigate the flow around a cylinder at Reynolds number = 3900. They concluded that low mesh resolution in the shear layer causes limited recirculation lengths values and vice versa. At Reynolds number = 5800, Shao and Zhang [22] compared the Reynolds-averaged Navier–Stokes (RANS) and large eddy simulation results (same sub-critical regime). The momentum equations were solved using a second-order upwind scheme and bounded central differencing scheme. Khan et al. [23] used LES and Smagorinsky SGS model to numerically simulate the flow over cylinder at Reynolds number = 3900. The simulation was run for 60 non-dimensional time steps before the time statistical data were collected to ensure that the flow was free of initial conditions and completely formed. The data was collected for 30 vortex-shedding periods. Feng et al. [5] uses LES model to compute hydrodynamic coefficients, pressure distribution, velocity profiles, Reynold’s stress distribution in wake, flow separation and recirculation length. The aim of this paper is to use a benchmark problem to compare the performance of the Open FOAM sub grid model quantitatively, as well as to address some key factors that affect predictive performance. Korinek et al. [7] used LES code and the Smagorinsky—Lilly subgrid-scale model to identify the effect of spanwise length and mesh resolution on measuring recirculation length and angle of separation around a fixed structure at Reynolds number = 3900. Most recently, Korinek et al. [9] used partially average NS-bridging technique to investigate the flow over same Reynolds number at different ratio of resolved and unresolved turbulent kinetic energy. A shorter recirculation length is obtained at low Reynolds number due to earlier transition. Filipe et al. [24] examined the modelling accuracy of distinct RANS equations and SRS method at Re = 3900. They concluded that SRS model is more accurate than RANS. Jiang and Cheng [25] investigated the flow around cylinder at Re = 400 to 3900 using opensource tool with emphasize on generalize mesh density, domain size and other parameters. Lekkalla et al. [10] performed two-dimensional numerical simulations at Reynold number of 3900 for rotating cylinder using k-e model. They investigated the vortex patterns and drag coefficients in the wake of the cylinder. Tian and Xiao [26] recently studied flow around cylinder using LES with conclusion that weak production rates due to shear layers delay the downward movement of the mean flow, resulting in a longer recirculation region. Most recently [27–31], the flow around cylinder (in laminar and turbulent regime) numerically and analytically has been investigated by numerous researchers for better understanding of the unsteady behavior [18, 29, 32–39].

Current study, which is an extension of the previous study (Khan, Ibrahim, Ali, et al. 2019; Khan, Ibrahim, Bin Mohamad Badry, et al. 2019), the impact of spanwise length (0.5D, 1D, 2D, πD, 4D, 8D to find optimum spanwise length), mesh resolution in spanwise direction (1 to 80 elements to find optimum mesh design) on the recirculation length, angle of separation, hydrodynamic coefficients and wake characteristics in detail at Re = 3900. Earlier the study was performed with spanwise length 4D-8D only.

## Computational domains, boundary conditions and mesh

Fluid flow around cylinder is highly dependent on the flow domain size. In the past studies, as referred earlier, size of the domain varies from 15D to 70D in crossflow (Y) and streamflow (X) directions. To resolve the wake region and boundary layers, grid points are being clustered in wake region and over the cylinder. In past studies, range of crossflow (L_y_) domain is kept between 80D to 20D, while range of streamflow (L_x_) domain is usually kept from 40D to 20D. In several cases, mesh is designed in such a way that it is divided into several regions i.e., O-Grid meshing is surrounded over cylinder and remaining region will then meshed using structured method. In the current study, domain size of 40D×20D (inflow x cross flow) is used, whereas the spanwise length is varied from 8D to 0.5D.

**Fig 1:**
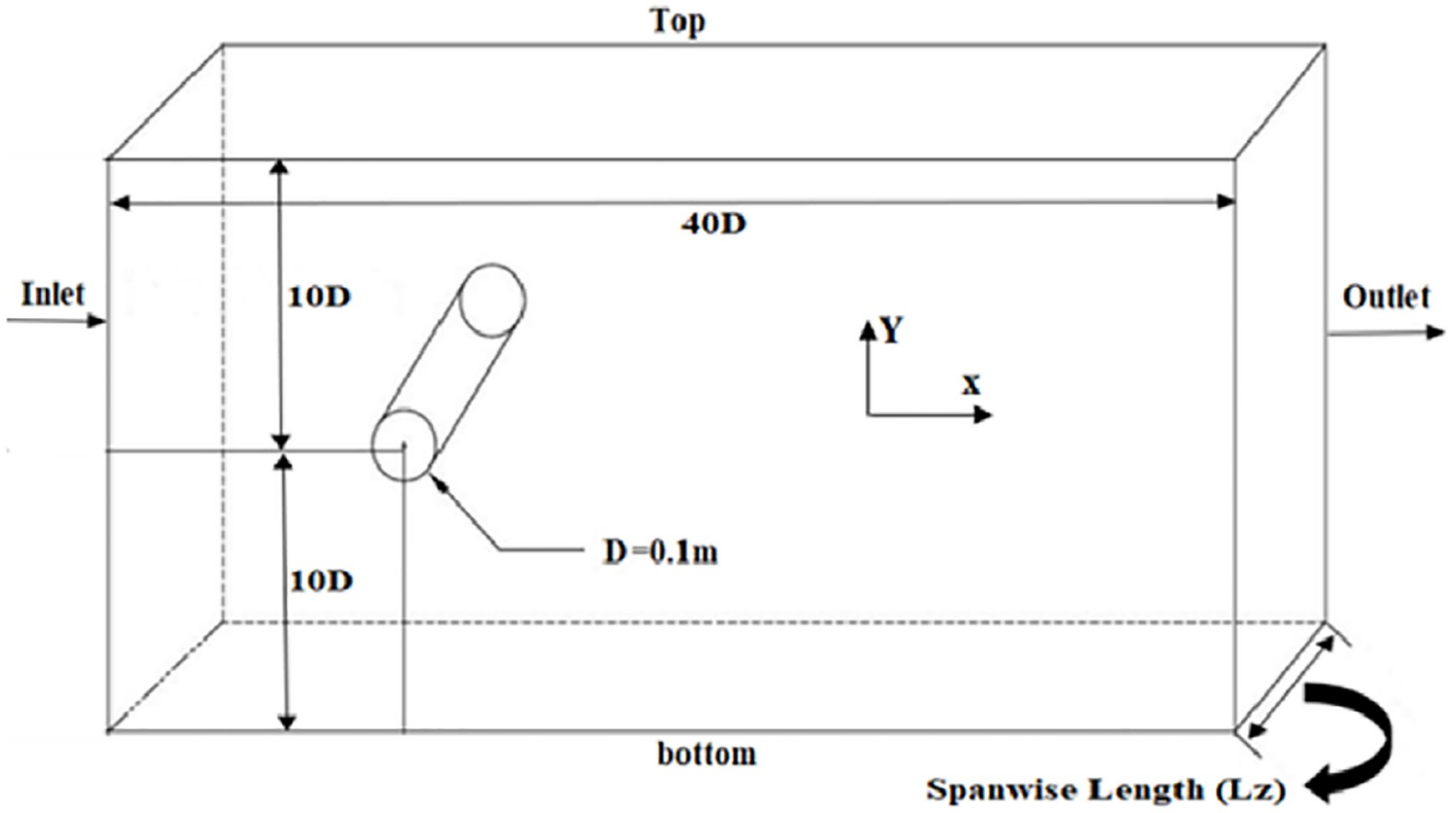
CAD model and boundary conditions.

Comparatively, a small variation in results are observed while varying the spanwise length from 4D to 8D [15, 19]. There is only 1% influence on solution by increasing spanwise length beyond 4D [40]. In order to minimize cylinder response effects, blockage ratio of 5% (cylinder diameter / domain width) is suitable [41], [4, 42]. Due to this reason, computational domain size of 40D×20D (streamflow ×crossflow) with varying spanwise lengths (8D, 4D, πD, 2D, 1D, 0.5D) is used in the current study. Flow parameters are highly influenced by aspect ratio. Therefore, boundary conditions are periodically assigned to bottom and top of the wall so they can reduce the effect of impact ratio on the numerical results. Left side inlet boundary of the domain is placed 10D from center of the cylinder. Right side outlet boundary of the domain is placed 30D from center of the cylinder as shown in Fig 1. Lower and upper walls of this computational domain is 10D from center of the cylinder and are symmetric in nature. In spanwise direction, periodic boundaries are assigned with multiple spanwise lengths (8D, 4D, πD, 2D, 1D, 0.5D).

**Fig 2:**
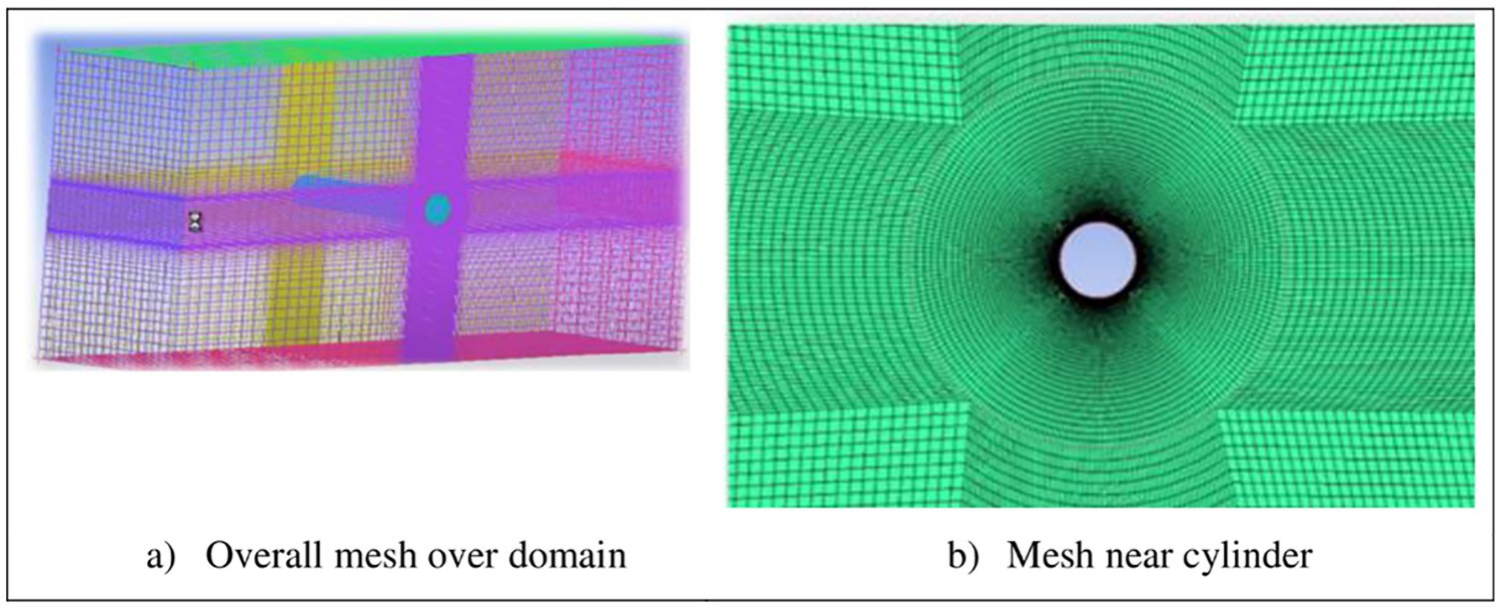
Mesh details.

Velocity at inlet is 0.6 m/s, which is on right side of domain, maintaining Reynolds number Re = 3900 by keeping diameter of cylinder D = 0.1m, viscosity = 0.000016 kg/ms, density = 1.04 kg/m^3^. At the outflow border, a static reference pressure of 0 Pa is applied on average. A symmetric wall condition is applied to both upper and lower walls of flow domain. To investigate the boundary layer separation and vortex generation phenomenon, a no-slip condition is given to cylinder wall. The modeling and analysis are performed using ANSYS tool (design modeler and ICEM CFD respectively). All the meshes are designed using structured method of meshing and the computational domain is then divided into number of regions in a manner that O-Grid near the boundary wall of cylinder and then structured meshed away from the cylinder, as shown in Fig 2. Rectangular domain is used in all numerical studies. Greifzu [6] concluded that, value of y^+^ must be smaller or equal to unity in order to ensure the proper resolutions of grids near the wall of cylinder. In all case studies, y+ value equal or less than 1 is maintained near the cylinder wall. First node is placed at 0.002D in all numerical studies, in order to completely ensure y^+^ less than unity. Fig 2 gives details of overall mesh design and view near the wall of cylinder respectively. Multiple meshes are designed with different spanwise lengths and in some cases, study with different mesh resolution is performed in order to validate previous study. Table 1 review the mesh type used in literature to perform flow around cylinder study.

**Table 1:** Mesh details and boundary conditions used in literature.

	N_T_×10^6^	Mesh type	L_x_ × L_y_	L_z_
[15]	62	O-Grid	25D×20D	4D-8D
[14]	45.8	Hybrid	20D×20D	πD
[12]	7.7	Hybrid	20D×20D	πD
[13]	5.76	O-type	50D	πD
[43]	13.5	Hybrid	32D×16D	4D
[44]	5.5	O-type	35D	πD
[4]	1.7	Hybrid	24D×8D	10D
[45]	6	Hybrid	30D×20D	4D
[46]	1.04	Hybrid	32D×16D	3.288D
[11]	1.74	O-type	30D	πD
[7]	2.031	Hybrid	40D×20D	4D
[7]	3.064	Hybrid	40D×20D	4D
[7]	4.063	Hybrid	40D×20D	8D

## Results and discussions

Table 2 shows case studies (A to X) with spanwise length ranging from 8D to 0.1D (8D, 4D, πD, 2D, 0.5D and 0.1D) and mesh density ranging from 0.0125 to 2. In all these case studies, number of elements on cylinder circumference (N_c_) × number of elements on cylinder in radial direction (N_R_) are 240×60 respectively. 240x60 is taken from the study of Khan et al. [7], who concluded that reduction in number of circumferential and radial nodes will overestimate the value of C_d_, reduction in recirculation length, and a delay in flow separation.

**Table 2:** Results comparison with grid variation in spanwise direction.

Case	N_T_×10^6^	L_z_	N_z_	M_z_ = L_z_/N_z_	N_c_×N_R_	C_d_	St	ϴ_s_	L_r_/D
**A**	2.51	8D	40	0.2	240×60	1.03	0.21	89.19	1.42
**B**	4.90	8D	80	0.1	240×60	0.98	0.21	87.66	1.70
**C**	9.813	8D	160	0.05	240×60	0.98	0.203	87.66	1.70
**D**	1.316	4D	20	0.2	240×60	1.0	0.187	89.19	1.11
**E**	2.51	4D	40	0.1	240×60	0.98	0.201	87.66	1.70
**F**	4.9033	4D	80	0.05	240×60	0.9866	0.200	87,66	1.70
**G**	1.0171	πD	15	0.2	240×60	1.0413	0.182	89.19	1.1132
**H**	1.97	πD	31	0.1	240×60	0.96	0.202	87.66	1.71
**I**	2.51	πD	40	0.0785	240×60	0.96	0.202	87.66	1.71
**J**	0.17	2D	1	2	240×60	1.42	0.198	104.44	0.38
**K**	0.41	2D	5	0.4	240×60	1.22	0.199	95.29	0.72
**L**	0.71	2D	10	0.2	240×60	1.04	0.201	89.19	1.36
**M**	1.31	2D	20	0.1	240×60	0.97	0.203	87.66	1.70
**N**	2.51	2D	40	0.05	240×60	0.97	0.197	87.66	1.70
**O**	0.41	1D	5	0.2	240×60	1.12	0.194	90.73	1.10
**P**	0.71	1D	10	0.1	240×60	1.01	0.212	87.66	1.60
**Q**	1.01	1D	15	0.06	240×60	1.00	0.212	87.66	1.60
**R**	2.51	1D	40	0.025	240×60	0.99	0.212	87.66	1.60
**S**	0.17	0.5D	1	0.5	240×60	1.59	0.198	101.3	0.32
**T**	0.29	0.5D	3	0.166	240×60	1.05	0.199	89.19	1.34
**U**	0.35	0.5D	4	0.125	240×60	1.07	0.198	89.19	1.25
**V**	0.41	0.5D	5	0.1	240×60	1.00	0.197	87.66	1.42
**W**	1.31	0.5D	20	0.025	240×60	1.02	0.215	87.66	1.42
**X**	2.51	0.5D	40	0.0125	240×60	1.04	0.205	87.66	1.42

In case studies (A to I) having spanwise length (Lz) of 8D, 4D and πD, it is clearly observed that results are well converged when the Mz is 0.1. Further improvement in Mz value (by increasing the number of elements in spanwise length) is only resulting in increasing computational cost. It is also found that drag coefficient and St number are less sensitive compared to separation angle and recirculation length. Even with higher value of Mz = 0.2 (Case A, D and G), drag coefficient and St number are well captured, however, deficiency in flow separation angle (ϴs) and recirculation length (Lr/D) is observed. Higher value of ϴs and shorter Lr/D is observed when the value of Mz is increased. It is concluded form case studies (A to I) that ϴs and Lr/D should be the focused more to get accurate result.

In case studies (J to N) having constant spanwise length of 2D whereas the number of elements in spanwise direction are varying from 1 to 40, resulting in mesh density (length/NO. of elements) ranging from 0.05 to 2. It is clearly observed from the result of case J that coarse mesh (1 element in spanwise direction = Nz) does not capture the flow behavior. Higher value of hydrodynamic coefficients, shorter recirculation length and delay in flow separation is observed. With increase in number of elements in spanwise direction, the results improve and converge. In case M, where number of elements are 20 (or Nz = 0.1) the hydrodynamic coefficients, strouhal number, recirculation length and angle of separation are computed well. Further increase in number of elements only results in increasing the computational costs. These case studies (J to N) show that having spanwise length of 2D, the results are converged at mesh density of 0.1. This behavior is observed at spanwise length of 8D, 4D, πD, and 2D having total number of elements of 4.90, 2.51, 1.97 and 1.31 million, respectively.

Additional studies are performed to further investigate the impact of spanwise length and mesh density on flow behavior. Further reduction in spanwise length (1D and 0.5D) with mesh density ranging from 0.2 to 0.025 have been investigated (Case J to Case X). Shorter recirculation length and delay in flow separation is observed in all the cases, irrespective of the mesh density, with spanwise length less than 2D. Although, drag and Strouhal number is well captured at high mesh density cases, however, the most sensitive parameters like recirculation length and flow separation angle are not converged. From all these studies, it is observed that minimum spanwise length should be equal or greater than 2D in order to well capture the hydrodynamic coefficient, recirculation length, flow separation and Strouhal number. Furthermore, at spanwise length of 2D, the mesh density should be kept equal to or higher than 0.1.

Table 3 show comparison between experimental [14], numerical [7, 13, 16, 20, 40], and current results (spanwise length 8D, 4D, πD, 2D, and 0.5D with mesh density of 0.1.

**Table 3:** At Re = 3900, comparison between numerical and experimental results.

Case	C_d_	ϴ_s_	Lr/D	St
[47], **experiment**	0.98±0.005	----	1.33±0.2	0.215±0.005
[14], **experiment**	---	---	1.560.21	0.21
**Laurenco and shih, experiment**	0.99	86	1.19	0.22
[48], **experiment**	0.98±0.005	---	---	0.21
[16], **DNS**	---	---	1.47	0.203
[49]	0.88	911	1.04	0.250
[13], **LES**	0.97	89	1.67	0.209
[12], **LES**	1.15	86.5	1.02	0.215
[40], **LES**	1.02	86	1.49	0.207
[50], **LES**	0.99	---	1.37	0.212
[7], **Case D, LES**	0.98	86.18	1.68	0.218
[7], **Case L, LES**	0.986	86.18	1.70	0.205
[7], **Case O, LES**	0.982	86.18	1.73	0.21
**Present Case A, 8D, LES**	0.98	86.77	1.70	0.21
**Present Case E, 4D, LES**	0.98	86.77	1.70	0.201
**Present Case H, πD, LES**	0.966	86.77	1.71	0.202
**Present Case M, 2D, LES**	**0.98**	**86.77**	**1.70**	**0.203**
**Present Case P, 1D, LES**	1.012	86.77	1.61	0.212
**Present Case W, 0.5D, LES**	1.0	86.77	1.43	0.204

**Fig 3:**
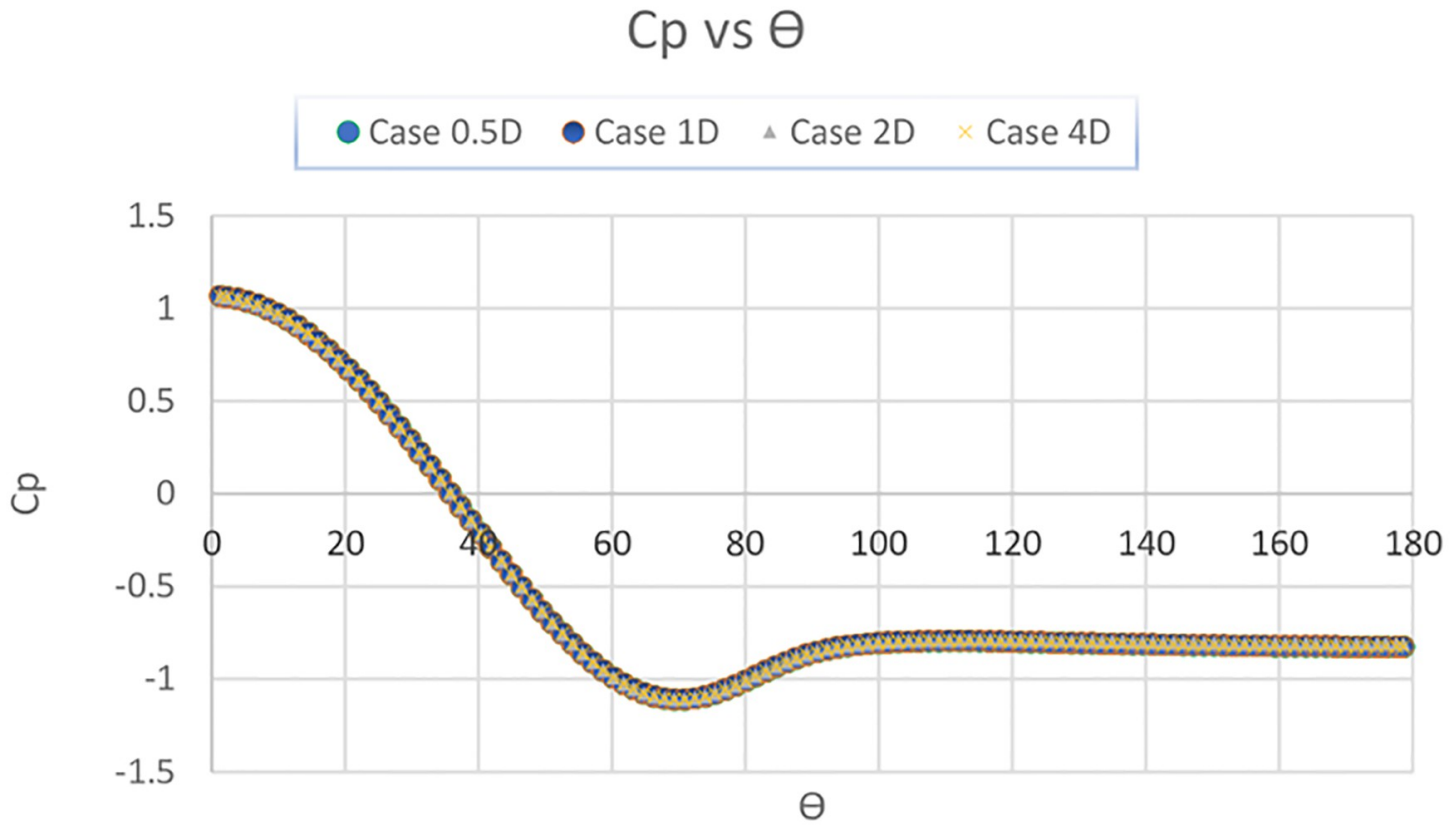
Coefficient of pressure (Cp) vs Angle (ϴ).

Fig 3 depicts pressure distribution over the cylinder surface. This research demonstrates that the findings are in strong accordance with the experimental and numerical results.

**Fig 4:**
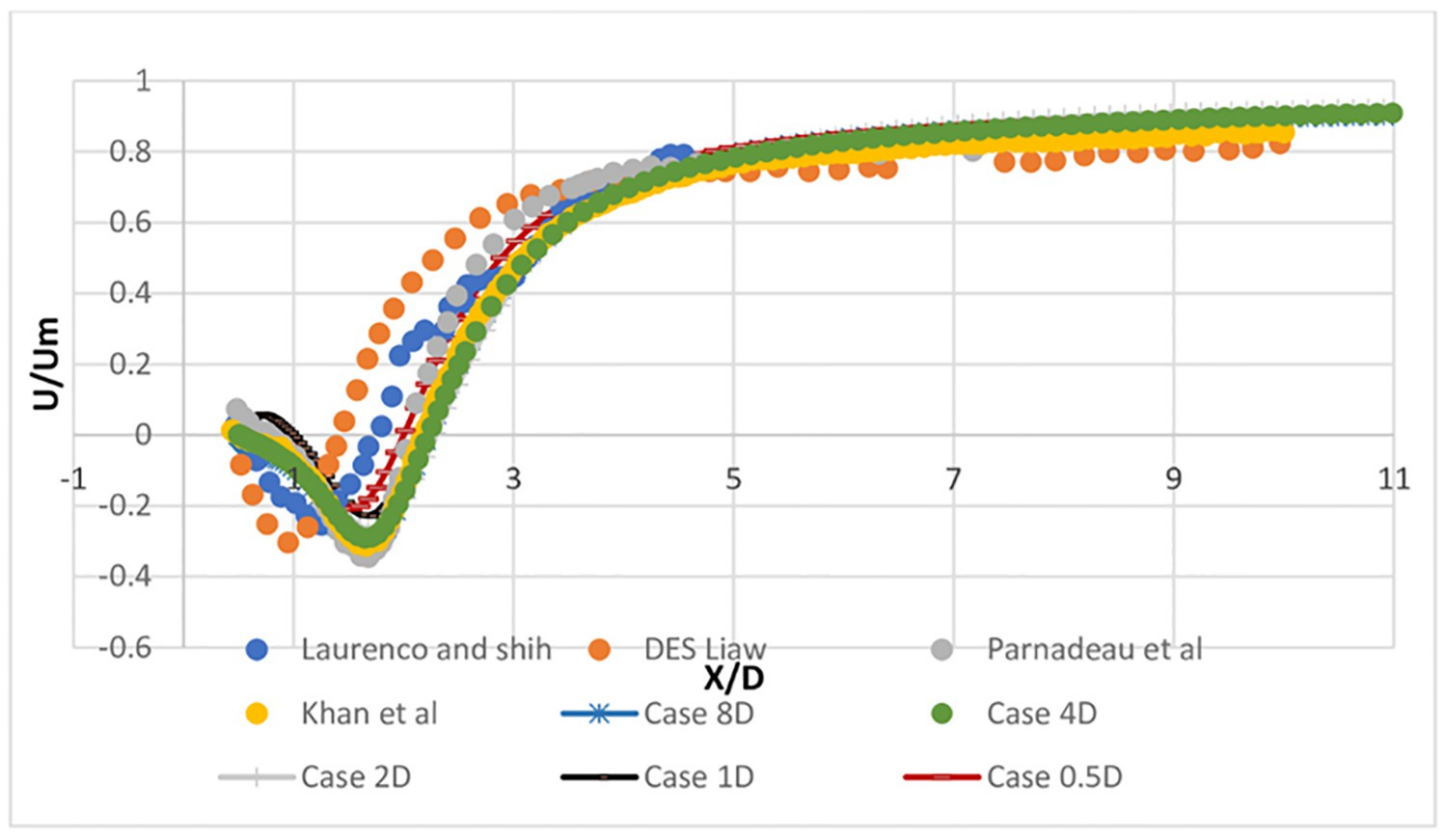
Mean stream velocity profile at centerline, behind the cylinder.

**Fig 5:**
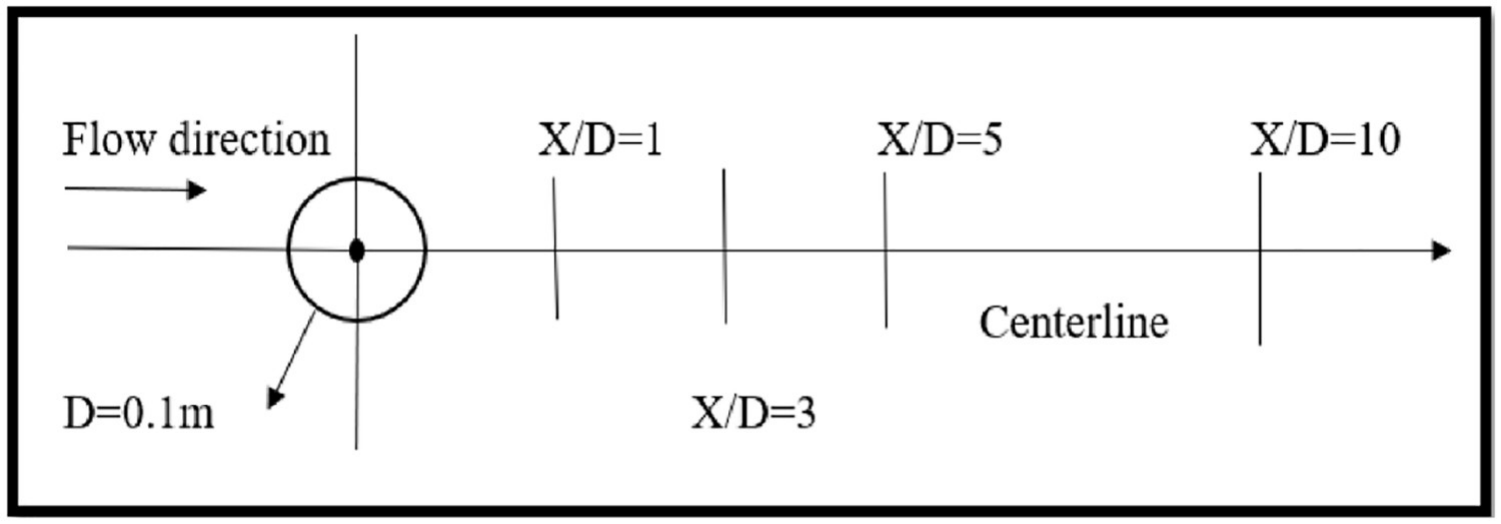
Vertical profiles and centerline sketch behind the cylinder.

**Fig 6:**
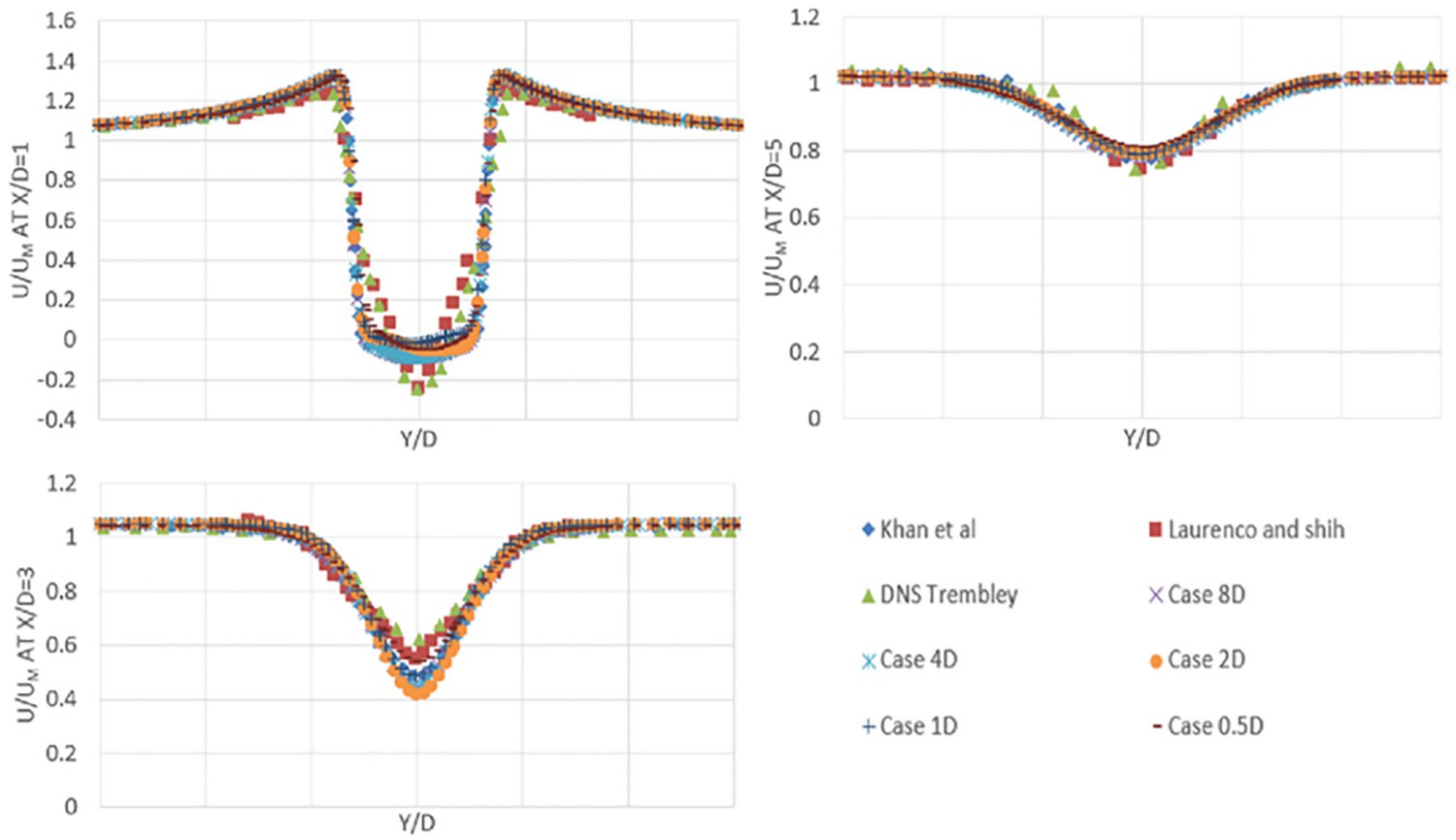
Comparison of mean stream velocity profiles of flow over cylinder at X/D = 1,3 and 5 for present study and other numerical and experimental results.

The mean streamwise velocity profiles in the wake of the cylinder are shown in Fig 4. The mean velocity is calculated from the cylinder’s centerline to a 10-diameter distance behind the cylinder, as shown in Fig 5. In Fig 6, the results show that the detached eddy simulation (DES) has a slightly shorter recirculation length in comparison with experimental results. There is a small disparity between the experimental results and the findings of this research study. This disparity may be due to Lourenco and Shih’s usage of the PIV process, where an external disruption causes the separating shear layer to transition early [21]. It is also observed that coarse mesh in spanwise direction result in v-shape profile of mean streamwise velocity which is improved to U-shaped with improvement in mesh density. Overall, this research study validates previous numerical and experimental results. This LES case study’s recirculation length matches well with Khan [7] numerical results.

Fig 6 compares the mean streamwise velocity profiles in the wake region to previous studies (at x/D = 1, x/D = 3, and x/D = 5). The data ranges from y/d = –3 to y/d = 3. By dividing the mean streamwise velocity by the inlet velocity, the mean streamwise velocity is normalized. Present results agrees well with Khan [7] results. Also, there is a slight difference in peak at x/d = 1, this discrepancy may be attributable to Lourenco and Shih’s different experimental methods, where some external disruption will cause early transition and shear layers separation. At x/d = 1, a U-shape profile is observed, indicating that the current data are reliable and accurate. When the grid resolution is coarse enough in the spanwise direction, a V-shaped profile is observed, according to Khan [7] and Kravchenko and Moin [21].

**Fig 7:**
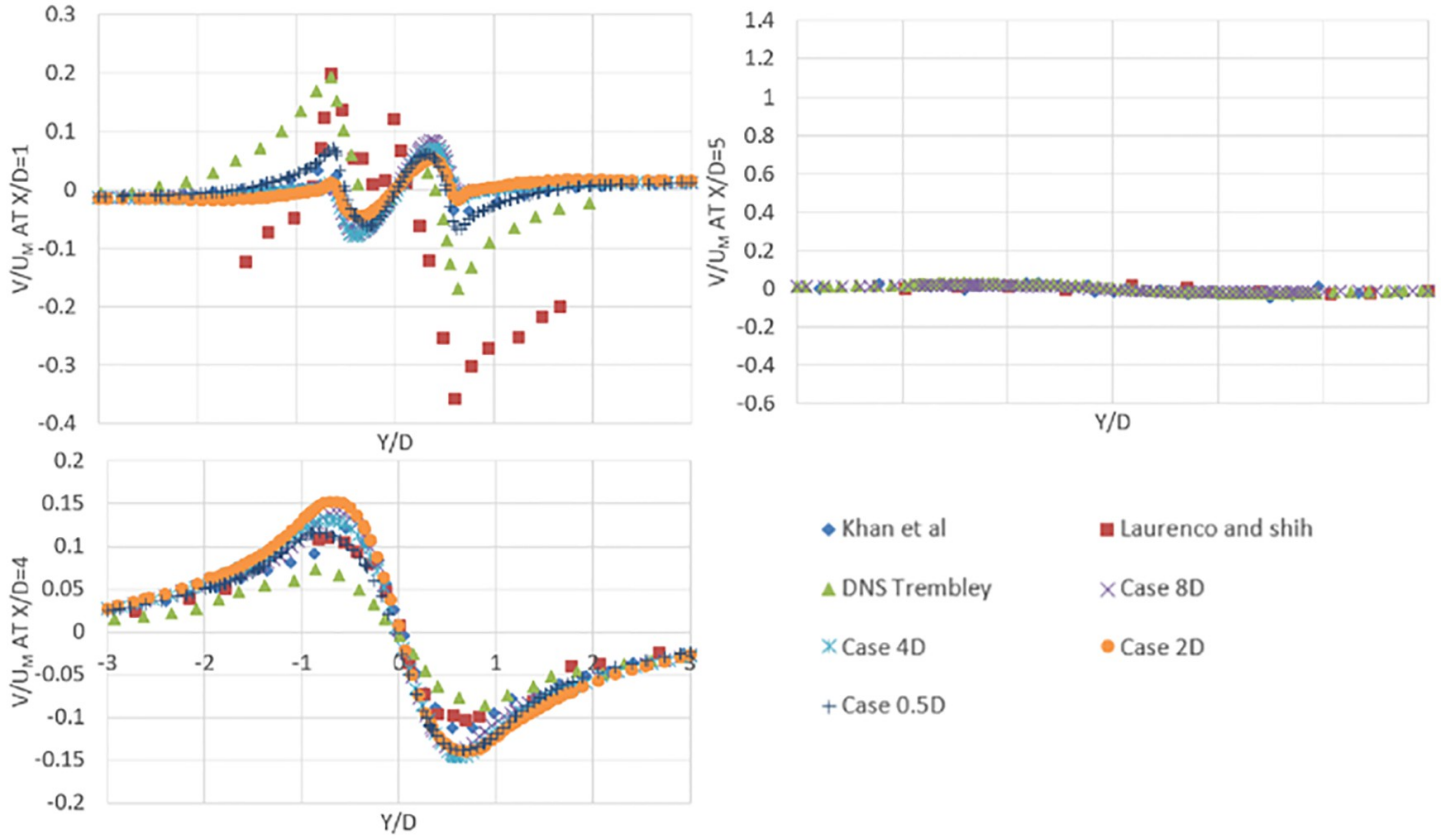
Comparison of mean crossflow velocity profiles of flow over cylinder at X/D = 1,3 and 5 for present study and other numerical and experimental results.

At x/D = 1, x/D = 3, x/D = 5, the mean crossflow velocity profiles in the wake region are shown in Fig 7. There is a difference between present study results and Lourenco’s and Shih’s experimental and Trembley [12] results in the wake region near the cylinder at x/D = 1. However, the current LES study is in accordance with the profile of the mean crossflow velocity component of simulations by Khan [7]. This LES analysis captures the results well, away from the cylinder. Norberg [47] investigated the wake flow near a cylinder with various geometrical parameters and found that the spanwise end condition has a major impact on the onset shear layer instability. The periodic boundary condition is used in simulations in the spanwise faces, so the aspect ratio near the cylinder wake has no effect on the results. However, near the wake, a grid independence analysis is needed to obtain accurate results.

## Conclusions

In this study, flow around cylinder at Re = 3900 has been investigated using LES model, with main focus on optimization of spanwise length and mesh distribution. This study mainly extended the previous studies by analyzing the case studies from (8D to 0.5D) with mesh density in the range of (2 to 0.0125). The study concluded that irrespective of the spanwise length, mesh density should not be kept greater than 0.1. Mesh density greater than 0.1 result in delay in separation angle, shorter recirculation length and over-predicted value of hydrodynamic coefficients. It is also observed that coarse mesh in spanwise direction result in V-shape profile of mean streamwise velocity which is improved to U-shaped with improvement in mesh density. With mesh density equal to 0.1, it has been concluded that optimum spanwise length of 2D is able to extract the reliable results of hydrodynamic coefficients, Strouhal number, separation angle and recirculation length. It is also observed that further reducing the spanwise length results in shorter recirculation length, even with high mesh density in spanwise length. It is also concluded that drag coefficient and Strouhal number are comparatively less sensitive parameters as the change is spanwise length and mesh resolution have minor impact on the result.

## Abbreviations

C_d_: Drag coefficient
C_L_: Lift coefficient
LES: Large eddy simulation
L_x_: Length whereas subscript x, y and z represents the coordinate direction
M_z_: Mesh resolution whereas subscript represent coordinate
N: Number of elements and subscript R and C stand for radial and circumference
Re: Reynolds number
S_t_: Strouhal number
U: Inlet velocity
